# Gender representation in surgery: progress and challenges in recent years

**DOI:** 10.1097/JS9.0000000000000189

**Published:** 2023-03-03

**Authors:** Aashna Mehta, Andrew A. Wireko, Favour Tope Adebusoye, Pearl Ohenawaa Tenkorang, Muhammad J. Zahid, Anushka Pujari, Heli Patel, Zoya Morani, Luis Morales Ojeda, Ayush Anand, Stefania M. Arcila, Arda Isik

**Affiliations:** a Faculty of Medicine, University of Debrecen, Debrecen, Hungary; b Sumy State University, Sumy, Ukraine; c University of Ghana Medical School, Accra, Ghana; d Department of General Surgery, Hayatabad Medical Complex, Peshawar, Pakistan; e Royal College of Surgeons – School of Medicine, Dublin, Ireland; f Dr Kiran C Patel College of Allopathic Medicine, Nova Southeastern University, Davie, Florida; g Washington University of Health and Science, San Pedro, Belize; h Institute of Urology, University of Southern California, Los Angeles, California, USA; i BP Koirala Institute of Health Sciences, Dharan, Nepal; j Department of General Surgery, Istanbul Medeniyet University, Istanbul, Turkey

*Dear Editor*,

Women have played an important role in medical history for centuries. The first surgical procedure involving a woman occurred over 5000 years ago^1^. Before the 1970s, medical schools had an extremely low female enrollment (6%)^1^. Over the past decades, the proportion of female medical students has increased dramatically, with female medical students now outnumbering male medical students in developed countries such as the UK and the USA^1^. Nonetheless, there has been minimal progress in the representation of females in the field of surgery, with less than one-third of surgeons worldwide being female^1^. Several factors influence junior female doctors’ decision to pursue a career in surgery. Some women are put off by the notion that surgery is an “old boys’ network” requiring male strength.

The Association of Women Surgeons (AWS) reported that in the USA, women made up only 8% of professors, 13% of associate professors, and 26% of assistant professors of surgery in 2015. Women accounted for 13% of professors, 21% of associate professors, and 29% of assistant professors in 2017^2^. In the UK, female representation for each specialty was as ollows: ophthalmology had 49.7%, otolaryngology had 48.2%, pediatric surgery had 45.5%, plastic surgery had 42.2%, general surgery had 39.8%, urology had 31.6%, vascular surgery had 25.0%, neurosurgery had 24.7%, cardiothoracic surgery had 21.3%, and trauma and orthopedics had 20.6%, respectively^3^. Africa has been assumed to have the fewest female surgeons of any continent, despite the continent’s lack of proper data to accurately assess the problem.

Gender disparities in surgical research have been quite significant, particularly in academic surgery where leadership positions and other factors come into play. Over the last 50 years, the number of female surgeons has steadily increased. In fact, since 1993, the gender gap in surgical research and authorship has decreased over years^4^. However, there is still a significant gender gap in surgical research authorship. There may be a couple of reasons for this discrepancy, although it is unclear what the main cause may be. Vora *et al.* 4 discovered that males have higher rates of publication and longer research longevity in comparison to their female counterparts. Female surgeons are also much less likely to continue research and publishing for more than 5 years after their first publication^4^. This “research pipeline” as Vora quotes, is an important factor during academic review and allocating senior faculty or leadership positions^4^. This gender disparity may be due to the lower availability of academic resources and protected time for female surgeons during their surgical residency in comparison to male surgeons^4^. Furthermore, women physicians are significantly more involved in childcare, “emotional labor,” and other caregiving duties, which takes time away from their research and reduces their work hours. Lack of strong mentorships, in particular, could be a significant barrier for women in surgery.

Physician pay varies by specialty, gender, race, number of years in practice, type of practice, location of practice, and employee productivity. Multiple surveys have found that women earn significantly less than men in the field of medicine overall^5^. Considering the field of surgery, unadjusted income disparities would result from lower female research and clinical productivity, according to one set of theories. Home obligations, parenthood, a relative lack of mentorship, unequal distribution of institutional research funds and workspace, and varying preferences for work-life balance are a few examples of these issues.

There is also a notable disparity in the representation of women in leadership positions, with female surgeons accounting for less than 20% of permanent faculty and 7.7% of surgery chairs^6^. This may limit access to same-sex mentors, which may have a psychological and societal impact on women’s decision to pursue surgery.

To lessen the degree of this worldwide issue, several surgical societies are proactively developing interventions to increase the recruitment and retention of female surgeons. In the UK, female representation within surgery has increased since 1991 due to notable contributions of Women in Surgery (WinS)^7^. Numerous groups similar to WinS, including, but not limited to AWS, the Royal Australasian College of Surgeons, and Women in Surgery Africa (WISA) have aimed to tackle gender inequality in surgery in recent years^3^,^8^. Within the USA, the Association of American Medical Colleges (AAMC) has developed policies and standards regarding the development of formal programs to promote a culture that enhances female recruitment and encourages a strong network of women faculty^9^. Also, organizations such as the Gender Equity Initiative in Global Surgery aim to address gender disparities in the surgery workforce in low- and middle-income countries, through research, advocacy, and mentorship to achieve worldwide gender equity in surgery by 2030. While identifying various targets of intervention to enhance gender diversity in surgery, the most often cited action items include: fostering and mentoring women in their career advancement, promoting qualified women to visible leadership positions, spreading awareness regarding gender discrepancy, and identifying and eliminating discrimination in recruitment and hiring of residents and faculty surgeon^10^. Successfully implementing programs targeting these action items can remove barriers that women face to achieving a successful surgical career. In the context of Africa, the continent with the worst-case scenario, WISA was founded in 2015 to address these disparities^8^. WISA has established a successful mentorship program in each of its member countries to encourage female aspiring surgeons to pursue their dreams. The American College of Surgeons (ACS) has also committed to supporting WISA by educating and training young female surgeons with scholarships^8^.

To improve gender diversity in the surgical workforce, a number of well-planned strategies must be implemented. Female surgeon training programs should be made more flexible to give these women the opportunity to have a successful private life and also find a balance. The surgical culture should be changed, and gender stereotypes should be corrected, by educating and raising awareness about a culture that welcomes women. Female surgeons should also be encouraged to take on leadership roles to boost their confidence and maximize their potential. During surgical residency, women should have equal access to opportunities and resources, and they should be free to work without fear. As a result, rules and regulations should be enforced to protect female surgeons who are verbally abused, harassed, and threatened.

Furthermore, more investments, energy, and resources should be directed toward bridging the gap in research and publications by female surgeons to address the slower “research pipeline” for female surgeons. Equal opportunities and scholarships should be made available to young women aspiring to be surgeons, particularly those from low-income countries. Female surgeons, residents, and surgical student aspirants should be encouraged and supported to participate in women’s surgical societies and professional organizations. This can contribute to a more welcoming and supportive environment for women in the surgical workforce, as well as opportunities for networking, mentorship, and professional development.

Low- and middle-income countries, especially in African countries, should work with other international organizations and stakeholders to promote gender diversity in the surgical workforce. They can efficiently work through the implementation of policies and programs that will highly promote women’s inclusion and advancement in surgery. To increase gender diversity in the surgical workforce, a combination of active efforts and interventions at all levels is required.

## Ethical approval and consent to participate

Not applicable.

## Sources of funding

None.

## Authors’ contribution

A.M. conceptualized the ideas. All authors were involved in writing of initial draft, reviewed and edited the manuscript, read and approved the final version of the manuscript.

## Conflicts of interest disclosure

The authors declare that they have no financial conflict of interest with regard to the content of this report.

## Research registration unique identifying number (UIN)

Not applicable.

## Guarantor

Andrew A. Wireko.
